# The impact of extracerebral organ failure on outcome of patients after cardiac arrest: an observational study from the ICON database

**DOI:** 10.1186/s13054-016-1528-6

**Published:** 2016-11-14

**Authors:** Leda Nobile, Fabio S. Taccone, Tamas Szakmany, Yasser Sakr, Stephan M. Jakob, Tommaso Pellis, Massimo Antonelli, Marc Leone, Xavier Wittebole, Peter Pickkers, Jean-Louis Vincent, on behalf of the ICON Investigators

**Affiliations:** 1 Department of Intensive Care, Erasme Hospital, Université Libre de Bruxelles, Brussels, Belgium; 2 Department of Critical Care, Royal Gwent Hospital, Newport, Wales UK; 3 Department of Anesthesiology and Intensive Care, Uniklinikum Jena, Jena, Germany; 4 Department of Intensive Care Medicine, University Hospital Bern, University of Bern, Bern, Switzerland; 5 Anesthesia and Intensive Care, Santa Maria degli Angeli Hospital, Pordenone, Italy; 6 Department of Intensive Care and Anesthesiology, Università Cattolica del Sacro Cuore, Rome, Italy; 7 Department of Anesthesia and Intensive Care, Hôpital Nord, AP-HM Aix Marseille Université, Marseille, France; 8 Critical Care Department, Cliniques Universitaires St Luc, UCL, Brussels, Belgium; 9 Department of Intensive Care, Nijmegen Institute for Infection, Inflammation and Immunity, Radboud University Nijmegen Medical Center, Nijmegen, The Netherlands; 10 Department of Anaesthetics, Intensive Care and Pain Medicine, Division of Population Medicine, Cardiff University, Cardiff, UK

## Abstract

**Background:**

We used data from a large international database to assess the incidence and impact of extracerebral organ dysfunction on prognosis of patients admitted after cardiac arrest (CA).

**Methods:**

This was a sub-analysis of the Intensive Care Over Nations (ICON) database, which contains data from all adult patients admitted to one of 730 participating intensive care units (ICUs) in 84 countries from 8–18 May 2012, except admissions for routine postoperative surveillance. For this analysis, patients admitted after CA (defined as those with “post-anoxic coma” or “cardiac arrest” as the reason for ICU admission) were included. Data were collected daily in the ICU for a maximum of 28 days; patients were followed up for outcome data until death, hospital discharge, or a maximum of 60 days in-hospital. Favorable neurological outcome was defined as alive at hospital discharge with a last available neurological Sequential Organ Failure Assessment (SOFA) subscore of 0–2.

**Results:**

Among the 469 patients admitted after CA, 250 (53 %) had had out-of-hospital CA; 210 (45 %) patients died in the ICU and 357 (76 %) had an unfavorable neurological outcome. Non-survivors had a higher incidence of renal (43 vs. 16 %), cardiovascular (56 vs. 45 %), and respiratory (62 vs. 48 %) failure on admission and during the ICU stay than survivors (all *p* < 0.05). Similar results were found for patients with unfavorable vs. favorable neurological outcomes. In multivariable analysis, independent predictors of ICU mortality were renal failure on admission, high admission Simplified Acute Physiology Score (SAPS) II, high maximum serum lactate levels within the first 24 h after ICU admission, and development of sepsis. Independent predictors of unfavorable neurological outcome were mechanical ventilation on admission, high admission SAPS II score, and neurological dysfunction on admission.

**Conclusions:**

In this multicenter cohort, extracerebral organ dysfunction was common in CA patients. Renal failure on admission was the only extracerebral organ dysfunction independently associated with higher ICU mortality.

**Electronic supplementary material:**

The online version of this article (doi:10.1186/s13054-016-1528-6) contains supplementary material, which is available to authorized users.

## Background

Rates of sudden cardiac arrest (CA) vary around the globe, but it is estimated that the incidence is around 55 events per 100,000 person-years [1]. Overall outcomes remain poor, with less than 10 % of patients leaving the hospital alive with good neurological recovery [1]. Brain damage, exacerbated by global ischemia-reperfusion injury, is the leading cause of death [2–4]. Therapeutic hypothermia (TH) has been employed following return of spontaneous circulation (ROSC) to limit the extent of brain damage, although concerns remain regarding optimal temperature levels, target populations, and duration of cooling [5, 6]. The so-called “post-cardiac arrest syndrome” links ischemia-reperfusion injury with brain damage, myocardial dysfunction, and a systemic inflammatory response that has remarkable similarities to that of sepsis and may result in the development of multi-organ failure (MOF), regardless of whether or not TH is used [7].

There are limited data available on the extent and prognostic value of extracerebral organ dysfunction after CA. In a recent report [8], almost all (96 %) of the 203 patients resuscitated after CA had some degree of organ dysfunction, in particular cardiovascular and respiratory impairments; two-thirds of these patients had dysfunction of at least two extracerebral organ systems. Only alterations in the cardiovascular and respiratory systems, as assessed by Sequential Organ Failure Assessment (SOFA) subscores, were independently associated with in-hospital mortality. Renal dysfunction has also been reported as an independent prognostic factor for mortality among CA survivors [9], although conflicting data have been reported [10]. There are almost no data on coagulation dysfunction in this setting, and only one study described the occurrence of hypoxic hepatitis, but not liver dysfunction, in 11 % of CA survivors, which was also associated with increased intensive care unit (ICU) mortality [11].

Information on whether extracerebral organ dysfunction influences the outcome of patients after CA could open new lines of research, in particular related to clinical management and development of strategies to prevent such complications. The objectives of this study were, therefore, to assess the incidence of extracerebral organ failure in patients resuscitated from CA and its impact on prognosis, both in terms of ICU mortality and neurological outcome. For this purpose, we analyzed data from a contemporary, international database of ICU patients—the Intensive Care Over Nations (ICON) audit [12].

## Methods

Full details of methodology have been provided previously [12] and a list of participating ICUs is given in Additional file 1 (Appendix 1).

### Participating centers

Participating centers were recruited by open invitation, through national scientific societies, international meetings, and/or individual contacts. Participation was voluntary with no financial reimbursement. The participating institutions obtained local ethical approval.

### Inclusion criteria

Each center prospectively collected data on all adult patients (>16 years of age) who were admitted to their ICU from 8–18 May 2012; patients who remained in the ICU for <24 h (i.e., for routine post-operative surveillance) or who were readmitted were not included. Data were collected daily during the ICU stay for a maximum of 28 days, using electronic case report forms through a secured web-based platform. Patients were followed up for outcome data until death or hospital discharge, whichever came first. Decisions regarding withdrawal of care were made according to local practices; patients in whom a decision was made to withdraw care were considered in the final analysis.

### Data collection

Demographics and comorbid diseases (including chronic obstructive pulmonary disease, solid or hematologic cancer, liver cirrhosis, heart failure, acquired immunodeficiency syndrome, chronic renal failure, immunosuppression, severe malnutrition, and insulin-dependent diabetes mellitus) were collected on admission. Clinical and laboratory data for the Simplified Acute Physiology Acore (SAPS) II [13] and the Acute Physiology and Chronic Health Evaluation (APACHE) II [14] scores were reported as the worst values within the first 24 h after ICU admission. Microbiological and clinical infections were reported daily. A daily assessment of organ function was performed using the SOFA score [15].

### Definitions

Most of the definitions have been provided elsewhere [12]. Briefly, infection was defined in accordance with the International Sepsis Forum definitions [16]. Sepsis was defined as the presence of infection with the concomitant occurrence of at least one organ failure (i.e., a SOFA subscore for the organ in question of >2) [17]. Septic shock was defined as sepsis complicated by cardiovascular failure. For the purposes of this sub-study, patients with “post-anoxic coma” or “cardiac arrest” listed as the reason for ICU admission were considered as having been admitted after CA. CA was considered to have occurred out-of-hospital (OHCA) if patients were admitted through the emergency department and/or by ambulance; all other patients were considered to have had an in-hospital CA (IHCA). Patients in whom the lowest body temperature during the first day after ICU admission was <34 °C were considered to have received TH. At the time of the ICON audit, the targeted temperature management (TTM) study [6], which suggested similar effects on outcome for a cooling strategy using 33 °C or 36 °C, had not yet been published and routine practice was to target a temperature of 32–34 °C [18].

Patients were considered as “comatose” (central nervous system (CNS)-SOFA >2) or “non-comatose” (CNS-SOFA 0–2) on admission. ICU mortality rates and overall neurological outcomes were collected: patients who were alive at hospital discharge and in whom the last recorded CNS-SOFA subscore was between 0 and 2 (corresponding to a Glasgow Coma Scale (GCS) score of 10–15) were defined as having a favorable neurological outcome; other patients (non-survivors and survivors with CNS-SOFA of 3–4, i.e., GCS <10) were defined as having an unfavorable neurological outcome.

### Statistical analysis

Data are expressed as mean ± SD, median (interquartile range) or count (percentage), as appropriate. For continuous variables, normality assumption checking was performed by inspection of residual and normal plots. Differences between groups were assessed using the analysis of variance (ANOVA), Kruskal-Wallis test, Student’s *t* test, Mann–Whitney test, χ^2^ test, or Fisher’s exact test, as appropriate. The occurrence of organ failure was also analyzed according to the location of the arrest (IHCA vs. OHCA), the geographical region (Africa, Europe, Asia, Oceania, and America), and the gross national income (GNI) per person (≤US$4035 was defined as low and lower-middle income; $4036–$12,475 as upper-middle income, and >$12,476 as high income) [12]. The time-courses of each SOFA subscore in survivors and non-survivors or in patients with favorable or unfavorable neurological outcome were analyzed using generalized estimating equation models. Multivariable logistic regression was used to identify independent predictors of ICU death and of unfavorable neurological outcome. Variables with *p* < 0.2 in the univariate analysis were considered in the multivariable analyses. Colinearity between variables was excluded prior to modeling. Interactions between explanatory variables were also checked. The deviance of the logistic regression model and deviance and partial residuals were used to check for model adequacy. All reported *p* values are two-sided and *p* < 0.05 was considered to indicate statistical significance. Data were analyzed using IBM® SPSS® Statistics software, version 22 for Windows (IBM, Armonk, NY, USA).

## Results

Among the 10,069 patients included in the ICON registry, 469 had post-anoxic coma (*n* = 62) or CA (*n* = 407) as the reported reason for ICU admission and were thus considered as having had a CA. The mean patient age was 66 (52–77) years, and 282 (61 %) patients were male (Table 1). The CA occurred out-of-hospital in 250 (53 %) patients. On admission, the median SAPS II score was 60 (46–75) and the SOFA score was 10 (7–13); 337 (72 %) patients were comatose. A total of 210 (45 %) patients died during the ICU stay and 357 (76 %) had an unfavorable neurological outcome; decisions to limit therapy were made in 170 (36 %) patients.

**Table 1:** Characteristics of the study population, according to ICU survival and neurological outcome at hospital discharge

	All patients (*n* = 469)	Survivors (*n* = 247)	Non-survivors (*n* = 210)	Favorable neurological outcome (*n* = 97)	Unfavorable neurological outcome (*n* = 357)
Age, years	66 (52–77)	65 (52–75)	68 (52–78)	67 (53–75)	66 (52–77)
Male, *n* (%)	282 (61)	149 (61)	126 (61)	60 (62)	222 (60)
Weight, kg	75 (65–87)	75 (66–90)	71 (61–83)	75 (65–90)	74 (65–85)
OHCA, *n* (%)	250 (53)	120 (49)	124 (59)	34 (35)	216 (58)
IHCA, *n* (%)	219 (47)	127 (51)	86 (41)	63 (65)	156 (42)
No co-morbidities, *n* (%)	209 (44)	101 (41)	108 (51)	49 (51)	163 (44)
COPD/asthma, *n* (%)	65 (14)	39 (16)	26 (12)	17 (17)	51 (14)
Heart failure, *n* (%)	72 (15)	30 (12)	42 (20)	10 (10)	62 (17)
Diabetes, *n* (%)	45 (10)	20 (8)	25 (12)	12 (12)	33 (9)
Cancer, *n* (%)	38 (8)	18 (7)	20 (10)	10 (10)	28 (8)
Chemotherapy, *n* (%)	9 (2)	4 (2)	5 (2)	1 (1)	8 (2)
Liver cirrhosis, *n* (%)	14 (3)	1 (1)	13 (6)	1 (1)	13 (3)
Chronic renal failure, *n* (%)	50 (11)	21 (9)	29 (14)	10 (10)	40 (11)
HIV, *n* (%)	1 (0)	–	1 (1)	–	1 (1)
Corticosteroids, *n* (%)	14 (3)	6 (2)	8 (4)	1 (1)	13 (3)
Medical admission, *n* (%)	373 (83)	189 (91)	184 (89)	70 (74)	303 (84)
SAPS II score on admission	60 (46–75)	53 (39–64)	71 (57–81)*	38 (29–49)	65(54–79)*
SOFA score on admission	10 (7–13)	8 (6–11)	12 (9–14)*	6 (3–8)	11(8–13)*
Infections					
Infection on admission, *n* (%)	112 (24)	60 (24)	52 (25)	20 (21)	92 (25)
Infection any time, *n* (%)	200 (43)	120 (49)	80 (38)	41 (41)	159 (43)
Septic shock on admission, *n* (%)	75 (16)	40 (16)	35 (17)	11 (11)	64 (17)
Septic shock any time, *n* (%)	133 (28)	74 (30)	59 (28)	20 (21)	113 (30)
Vasopressors on admission, *n* (%)	262 (57)	124 (50)	138 (66)*	42 (43)	220 (62)*
Mechanical ventilation on admission, *n* (%)	408 (87)	205 (83)	203 (97)	67 (69)	341 (92)*
Mechanical ventilation any time, *n* (%)	425 (91)	211 (85)	204 (97)	71 (73)	354 (95)*
Hemodialysis/CRRT on admission, *n* (%)	38 (8)	19 (8)	19 (9)	4 (4)	34 (9)
Hemodialysis/CRRT any time, *n* (%)	103 (23)	59 (24)	44 (21)	17 (17)	86 (23)
MAP maximum on first day, mmHg	99 (87–112)	100 (89–113)	97 (85–111)	100 (88–113)	98 (87–112)
MAP minimum on first day, mmHg	61 (50–70)	63 (55–71)	56 (45–68)	63 (55–79)	60 (49–70)*
pH maximum on first day, mmHg	7.40 (7.30–7.45)	7.41 (7.35–7.46)	7.37 (7.23–7.44)*	7.42 (7.36–7.47)	7.39 (7.28–7.45)*
pH minimum on first day, mmHg	7.25 (7.11–7.35)	7.29 (7.19–7.36)	7.18 (7.05–7.29)*	7.32 (7.23–7.38)	7.23 (7.10–7.33)*
PaCO_2_ maximum on first day, mmHg	45 (37–57)	44 (36–55)	46 (37–60)	45 (37–53)	45 (36–60)
PaCO_2_ minimum on first day, mmHg	34 (28–40)	34 (28–40)	33 (27–39)	35 (29–40)	33 (28–40)
PaO_2_ maximum on first day, mmHg	144 (98–229)	135 (94–197)	149 (101–265)	141 (95–190)	145 (99–234)
PaO_2_ minimum on first day, mmHg	77 (61–98)	77 (64–94)	75 (57–105)	78 (66–90)	76 (60–100)
PaO_2_/FiO_2_ maximum on first day	232 (144–344)	232 (151–337)	233 (132–361)	270 (161–374)	227 (143–343)*
PaO_2_/FiO_2_ minimum on first day	195 (132–288)	200 (149–294)	186 (120–285)*	218 (165–300)	192 (124–285)*
Lactate maximum on first day, mmol/L	3.6 (2.0–7.6)	2.6 (1.5–5.5)	5.8 (2.5–10.5)*	2.1 (1.2–5.5)	4.30 (2.3–8)*
ICU stay, days	4 (2–8)	5 (3–10)	3 (1–6)*	4 (2–6)	4 (1–8)
Hospital stay, days	8 (2–20)	17 (8–31)	3 (1–6)*	15 (8–22)	5 (2–16)*
Limitation of care, *n* (%)	170 (36)	40 (16)	126 (61)*	11 (11)	159 (44)*
ICU mortality, *n* (%)	210 (45)	–	210 (100)*	–	210 (59)
Hospital mortality, *n* (%)	247 (54)	37 (15)	210 (100)*	11 (11)	236 (67)*

Values are given as median (interquartile range) unless otherwise stated

Missing values: 12 for mortality; 5 for sex; 36 for weight; 23 for source of admission; 15 for vasopressors on admission; 15 for ICU stay, 16 for hospital stay

*p < 0.05, survivors versus non-survivors; favorable outcome versus unfavorable outcome

*COPD* chronic obstructive pulmonary disease, *CRRT* continuous renal replacement therapy, *FiO*_*2*_ fraction of inspired oxygen, *HIV* human immunodeficiency virus, *ICU* intensive care unit, *IHCA* in-hospital cardiac arrest, *MAP* mean arterial pressure, *OHCA* out-of-hospital cardiac arrest, *PaO*_*2*_ partial pressure of oxygen, *PaCO*_*2*_ partial pressure of carbon dioxide, *SAPS* Simplified Acute Physiology Score, *SOFA* Sequential Organ Failure Assessment

Mean arterial pressure, pH, and PaO_2_/FiO_2_ ratio on the first day were lower, and maximal lactate level higher, in non-survivors than in survivors (Table 1). Non-survivors also had higher SAPS II and SOFA scores on admission, and were more frequently treated with vasopressors. Similar patterns were found when patients with favorable and unfavorable neurological outcomes were compared (Table 1).

**Fig. 1:**
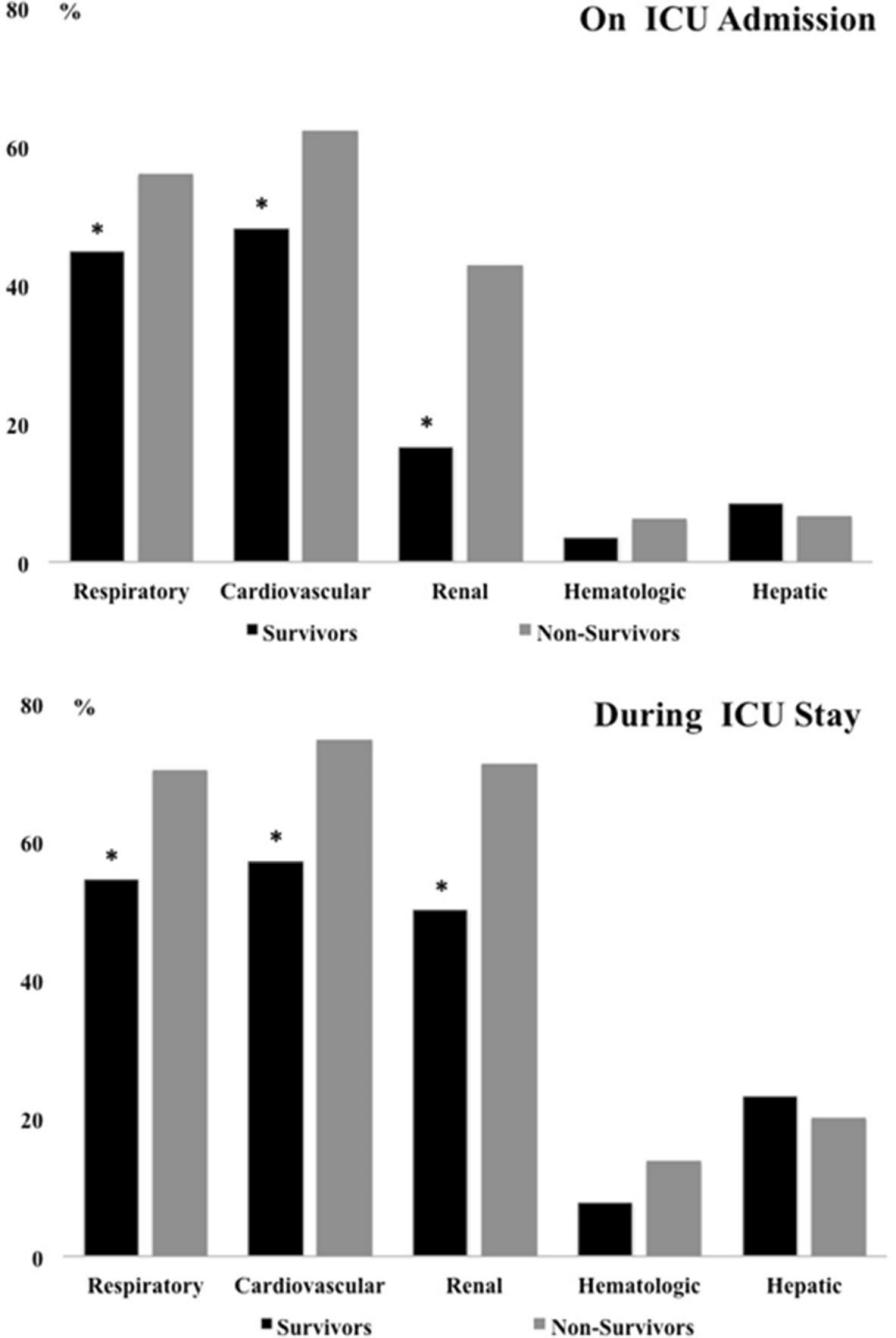
Occurrence of extracerebral organ failure on intensive care unit (*ICU*) admission (*upper panel*) and during the ICU stay (*lower panel*) in survivors and non-survivors. *p < 0.05

Non-survivors had a greater incidence of renal, respiratory, and cardiovascular failure than survivors on admission (43 vs. 16 %, 56 vs. 45 %, 62 vs. 48 %, respectively; all *p* < 0.05; Fig. 1) and at any time during the ICU stay (71 % vs. 50 %, 71 vs. 54 %, 75 vs. 57 %, respectively; all *p* < 0.05; Fig. 1). Similar patterns were found in patients with unfavorable and favorable neurological outcomes (Additional file 1: Figure S1). Patients with OHCA had a greater incidence of hematologic failure than those with IHCA, both on ICU admission (7 % vs. 2 %; *p* = 0.04) and during the ICU stay (14 % vs. 7 %; *p* = 0.009; Additional file 1: Figure S2); the occurrence of other organ failures was similar. There was considerable variability in the occurrence of organ failures in different geographical areas: patients from Oceania had the highest incidence of hepatic failure on admission, whereas respiratory failure was significantly more frequent in patients in Africa and Europe, and hematologic failure occurred more frequently in patients in Africa and Asia (Additional file 1: Figure S3). There were no statistically significant differences in the incidence of organ failure when patients were analyzed according to the GNI (Additional file 1: Figure S4).

**Fig. 2:**
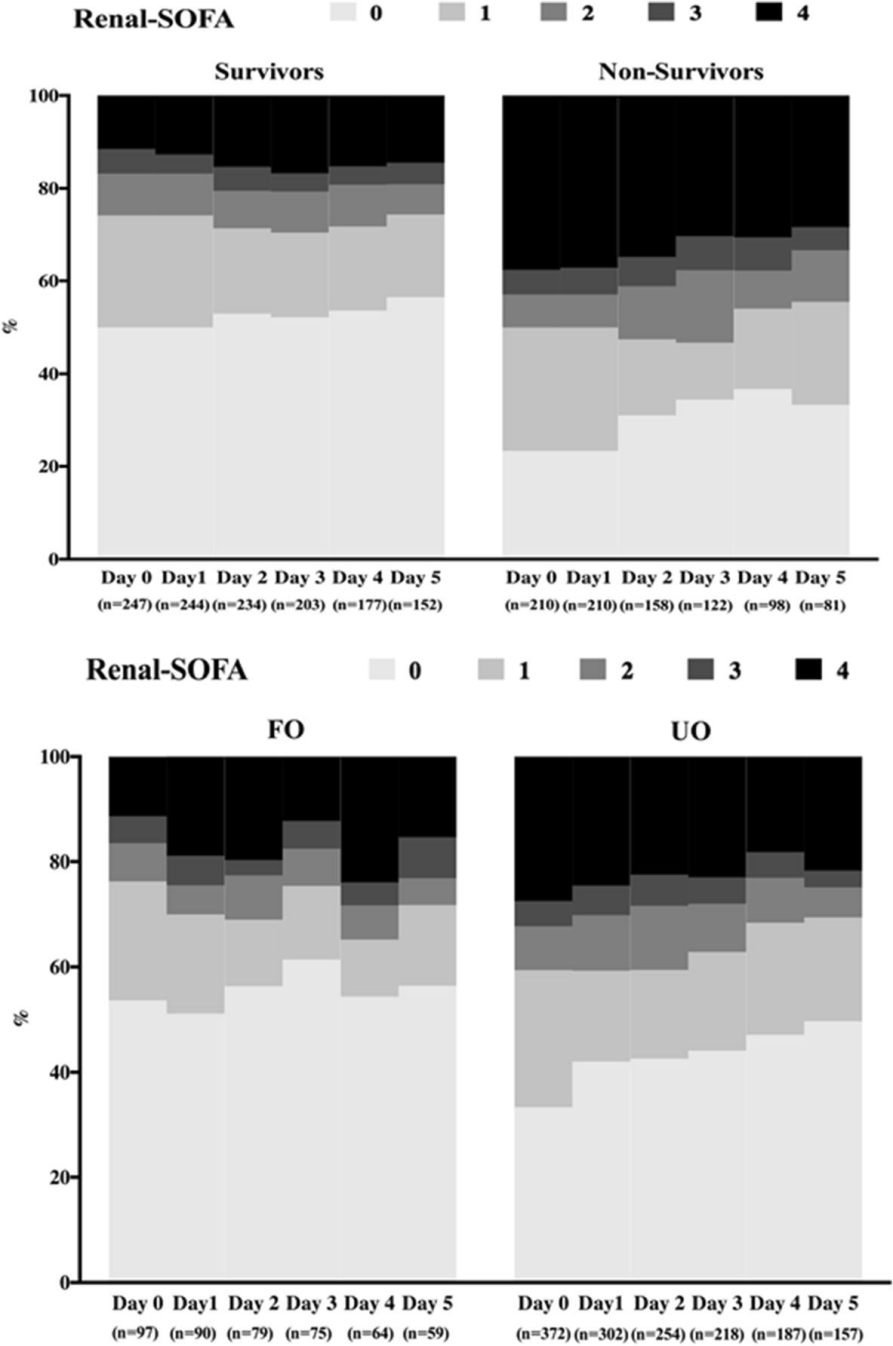
Time-course of renal-SOFA subscore in survivors and non-survivors (*upper panel*) and in patients with favorable (*FO*) and unfavorable neurological outcome (*UO*) (*lower panel*). *SOFA* Sequential Organ Failure Assessment

The time-course of several of the SOFA subscores differed in survivors and non-survivors. In particular, the hepatic-SOFA subscore increased over time in the non-survivors but not in the survivors (*p* = 0.015; Additional file 1: Figure S5), whereas CNS- and cardiovascular-SOFA subscores decreased over time in the survivors but not in the non-survivors (both *p* < 0.001; Additional file 1: Figures S6 and S7). The renal-SOFA subscore did not change over time in the survivors, but was higher in the non-survivors and decreased progressively over time (*p* < 0.001, Fig. 2). The time-course of respiratory- and hematologic-SOFA subscores was similar in survivors and non-survivors (Additional file 1: Figures S8 and S9). Renal-SOFA subscores decreased over time in patients with a favorable but not in those with an unfavorable outcome (*p* = 0.02; Fig. 2). Similar results were found for the CNS-SOFA subscore (Additional file 1: Figure S10). The analysis of other SOFA subscores showed a similar temporal trend between patients with favorable and unfavorable neurological outcome (Additional file 1: Figures S11–S14).

**Fig. 3:**
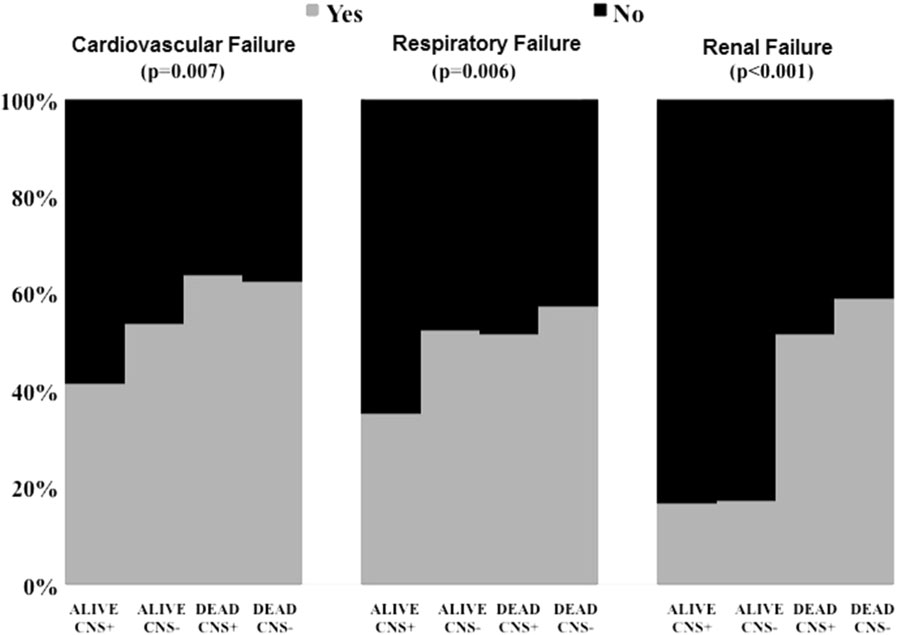
Occurrence of circulatory, respiratory, and renal failure in patients according to their outcome (alive or dead, with neurological recovery (*CNS+*) or persistent neurological impairment (*CNS–*))

Among the ICU survivors, 150 (57 %) patients had an unfavorable neurological outcome. Thirty-three (16 %) of the non-survivors had good neurological function (CNS-SOFA score of 0–2) before death (Additional file 1: Figure S15); however, the CNS-SOFA score on admission was also 0–2 in all these patients (0 in 19 patients, 1 in 3 patients and 2 in 11 patients); no patient who was comatose on admission and eventually died had improved neurological function during the ICU stay. These non-survivors with good neurological function more frequently had renal failure than did survivors (*p* < 0.001 versus survivors with good neurological function and versus survivors with poor neurological function); they also more frequently had cardiovascular failure (*p* = 0.026) and respiratory failure (*p* = 0.094) than the ICU survivors with good neurological function (Fig. 3).

In the multivariable analysis, renal failure on admission, high SAPS II score, high serum lactate levels within the first 24 h after ICU admission, and the development of sepsis were independent predictors of ICU mortality (Table 2). Mechanical ventilation on admission, high SAPS II score, and high CNS-SOFA score on admission were independent predictors of unfavorable neurological outcome (Table 2). None of the extracerebral organ failures occurring during the ICU stay were independently associated with ICU mortality or neurological outcome.

**Table 2:** Multivariable analysis to identify the independent predictors of ICU mortality and unfavorable outcome

Variable	*p* value	OR	95 % CI
Predictors of ICU mortality			
SAPS II score on admission	<0.001	1.047	1.025–1.069
Lactate max, mEq/L	0.004	1.093	1.029–1.161
Renal failure on admission	0.011	2.413	1.220–4.774
Severe sepsis during ICU stay	0.022	0.537	0.316–0.912
Predictors of unfavorable outcome			
SAPS II on admission	<0.001	1.107	1.077–1.138
MV on admission	0.020	3.787	1.234–11.628
CNS-SOFA on admission	<0.001	4.237	3.097–5.796

*CI* confidence interval, *CNS* central nervous system, *ICU* intensive care unit, *Lactate max* maximal lactate levels within the first 24 h after ICU admission, *MV* mechanical ventilation, *OR* odds ratio, *SAPS* Simplified Acute Physiological Score, *SOFA* Sequential Organ Failure Assessment

## Discussion

This international observational study showed that patients with a poor outcome after CA had a higher incidence of renal, cardiovascular, and respiratory failure on admission or during the ICU stay than did patients with good outcomes. Renal failure on admission was an independent predictor of ICU death, as was the severity of disease, high serum lactate levels, and the development of sepsis. However, extracerebral organ failure occurring later during the ICU stay did not significantly influence ICU mortality or neurological outcome.

Renal dysfunction has been reported in nearly 50 % of patients after CA, in particular in patients with pre-existing renal dysfunction, older age, longer duration of resuscitation, and the presence of shock [9, 10, 19–21]. Severe acute kidney injury (AKI) was associated with significantly higher mortality at 30 days after CA in one study [9], but AKI was not an independent predictor of mortality or poor neurological outcome in two others [10, 20]. Interestingly, despite the association of renal failure on admission with increased mortality, the rate of renal replacement therapy (RRT) was not different between any of the groups in our study. It is difficult to compare these findings as different confounders, including measured outcomes (e.g., hospital survival vs. neurological outcome), the use of TH, and the proportion of patients with OHCA, may have influenced the results. Moreover, evaluation of the impact of RRT on the prognosis of CA patients may also be influenced by local practice regarding renal support; for example, the use of RRT for correction of electrolyte disturbances and/or fluid overload in patients without overt AKI may be associated with better recovery and outcome than RRT initiated for severe renal failure. In addition, as a number of patients with extensive brain injury undergo limitation of life-sustaining therapies, regardless of the development of AKI, it is not always possible to assess the impact of AKI per se on outcome. In our study, renal failure was a more significant determinant of outcome than cardiovascular or respiratory dysfunction. Our data differ from those of Roberts et al. [8] who reported that extracerebral organ dysfunction was common after CA but found that only cardiovascular dysfunction and altered gas exchange were associated with outcome. However, that study [8] was conducted in a single US academic hospital over several years, whereas in our multicenter international audit, data were collected over a short time period. Moreover, Roberts et al. [8] did not record blood lactate levels and we observed that increased lactate concentrations after CA were significantly associated with ICU mortality. As high lactate concentrations are primarily a consequence of prolonged CA and/or severe subsequent hemodynamic impairment [22–24], lactate can be considered as an extracerebral variable that is predictive of poor outcome in these patients. Abnormalities in tissue perfusion occurring after CA may also potentially contribute to brain hypoperfusion and development of MOF [7]. Thus, monitoring of lactate in this setting may be of more value to assess the severity of tissue hypoxia than just blood pressure or cardiac output. Other studies have also shown the prognostic value of admission blood lactate levels after CA and of changes in lactate levels in the hours after CA [22–25].

In this large database, patients admitted after CA represented around 5 % of the ICU population, similar to values reported in other studies [26]. The SAPS II score was higher in patients with poor neurological outcome or those who died during their ICU stay than in the other patients, although others have reported that the SAPS and APACHE scores are not good prognostic tools in patients with CA [27, 28]. Conversely, the highest extracerebral SOFA score at 72 h after CA was independently associated with in-hospital mortality [8]. Admission factors that have been correlated with an increased SOFA score after CA include non-shockable rhythm, the amount of epinephrine used, use of TH, and elevated stress hormone levels [29]. In particular, the cardiovascular component of the SOFA score has been shown to accurately predict outcomes of patients with CA when combined with neurological examination [30]; our results suggest that the renal component should perhaps also be considered in prediction models.

Mechanical ventilation was one of the independent predictors of poor neurological outcome. Sutherasan et al. showed that high tidal volume and plateau pressure with lower positive end-expiratory pressure were associated with the occurrence of severe pulmonary complications during the ICU stay in CA patients [31], suggesting a potential role of ventilator settings on outcome. Moreover, patients receiving mechanical ventilation are more exposed to high oxygen levels or abnormal carbon dioxide concentrations, which have been shown to have a significant negative impact on brain recovery after post-anoxic injury [32, 33].

Not surprisingly, the severity of brain dysfunction on admission also predicted an unfavorable outcome. In a large cohort of CA patients in Japan, the initial GCS motor score was significantly associated with neurological outcome, as were age, bystander cardiopulmonary resuscitation (CPR), the time from collapse to ROSC, and pupil size [2]. Initial coma was also one of the most important determinants of outcome in another large database of CA patients [30].

Another interesting finding was that 16 % of the patients who eventually died had relatively preserved cerebral function during their ICU stay. In recent years, several studies have focused on identifying markers of poor neurological outcome as if mortality after resuscitated CA were exclusively associated with lack of neurological recovery [3, 4, 34]. Neurological recovery was often assessed 3 to 6 months after CA and all non-survivors were considered to have a poor neurological outcome. However, a significant proportion of CA patients die from protracted shock and MOF [4], which often occurs early after CA, before any possible neurological outcome assessment. In our study, patients with good neurological function after CA died from other reasons, in particular other organ failures. This finding highlights the importance of repeated neurological assessment of CA patients during the ICU stay because a single long-term assessment may underestimate the potential for neurological recovery in some patients.

This study has several limitations. First, the ICON database was not designed specifically to record data on post-CA disease, thus some key variables (e.g., initial rhythm, time to ROSC, or quality of CPR) were not captured, which limited the degree of adjustment for outcome analyses. Second, our cohort included patients with “post-anoxic coma” and “cardiac arrest” as the reason for ICU admission; it is possible that patients with cerebral injury without a CA (e.g., after hanging) may have been included in the "post-anoxic coma" category. Moreover, the delay between the occurrence of CA and the presence of neurological impairment was not available. Third, we focused on ICU and hospital mortality, but not on longer-term outcomes. In addition, the use of CNS-SOFA to assess neurological outcome in CA patients has several limitations and is not as accurate as other scores, e.g., the Rankin scale or the Cerebral Performance Scale (CPC), to quantify the extent of post-anoxic brain injury. Also, we could not evaluate cognitive dysfunction, which may reflect subtle post-anoxic injury. Fourth, given the substantial differences in post-resuscitation care in the countries included in the ICON database, patient management, including healthcare systems, resources and medical systems, was probably extremely variable among centers. Data about specific treatments (e.g., coronary angiography) were not available. Decisions regarding withdrawal of care certainly varied among regions. Nevertheless, this study is hypothesis-generating and may help to provide a focus on particular organ dysfunctions that should be adequately described in future prospective studies dealing with management and prognostication in such patients. Fifth, data on the duration of hypothermia and potential complications associated with TH were not available. Data on the development of fever were also not available. Sixth, data on “ventilator settings” during mechanical ventilation were not included in the analysis and we cannot comment on the roles of tidal volume, spontaneous breathing, and/or exposure to high-inspired oxygen fraction on the measured outcomes. Finally, the precise causes of death were not recorded.

## Conclusions

In this multicenter cohort of CA patients, 210 (45 %) patients died in the ICU and 357 (76 %) had an unfavorable neurological outcome. Extracerebral organ dysfunction was common, but renal failure on admission was the only extracerebral organ dysfunction independently associated with higher ICU mortality.

## Key messages

- Analysis of this large database revealed that renal, cardiovascular, and respiratory failure occurring on admission or during the ICU stay was more common in patients with an unfavorable outcome than in those with a favorable outcome.
- Renal failure on admission was the only extracerebral organ dysfunction associated with ICU mortality.
- Extracerebral organ failure occurring later during the ICU stay did not significantly influence ICU mortality or neurological outcome.

## Additional file

Additional file 1: Appendix 1. Alphabetical list of participating centers by region and country. **Figure S1.** Occurrence of extra-cerebral organ failure on ICU admission (upper panel) and during the ICU stay (lower panel) in patients with favorable (FO) and unfavorable (UO) neurological outcomes. **p* < 0.05. **Figure S2.** Occurrence of extracerebral organ failure on ICU admission (upper panel) and during the ICU stay (lower panel) in patients with in-hospital (IHCA) and out-of-hospital (OHCA) cardiac arrest. **p* < 0.05. **Figure S3.** Occurrence of extracerebral organ failure on ICU admission (upper panel) and during the ICU stay (lower panel) in patients according to geographical area. **Figure S4.** Occurrence of extracerebral organ failure on ICU admission (upper panel) and during the ICU stay (lower panel) in patients according to gross national income. **Figure S5.** Time course of hepatic-SOFA subscore in survivors and non-survivors. **Figure S6.** Time course of neurologic (CNS)-SOFA subscore in survivors and non-survivors. **Figure S7.** Time course of cardiovascular-SOFA subscore in survivors and non-survivors. **Figure S8.** Time-course of respiratory-SOFA subscore in survivors and non-survivors. **Figure S9.** Time-course of hematologic-SOFA subscore in survivors and non-survivors. **Figure S10.** Time-course of neurologic (CNS)-SOFA subscore in patients with favorable (FO) and unfavorable (UO) neurological outcome. **Figure S11.** Time-course of cardiovascular-SOFA subscore in patients with favorable (FO) and unfavorable (UO) neurological outcome. **Figure S12.** Time course of hematological-SOFA subscore in patients with favorable (FO) and unfavorable (UO) neurological outcome. **Figure S13.** Time-course of respiratory-SOFA subscore in patients with favorable (FO) and unfavorable (UO) neurological outcome. **Figure S14.** Time-course of hepatic-SOFA subscore in patients with favorable (FO) and unfavorable (UO) neurological outcome. **Figure S15.** Differences in the last available SOFA subscores in survivors (S) and non-survivors (NS). (PDF 1391 kb)

## Abbreviations

AKI: Acute kidney injury
APACHE: Acute Physiology and Chronic Health Evaluation
CA: Cardiac arrest
CNS: Central nervous system
CPR: Cardiopulmonary resuscitation
GCS: Glasgow Coma Scale
GNI: Gross national income
ICON: Intensive Care Over Nations
ICU: Intensive care unit
IHCA: In-hospital cardiac arrest
MOF: Multi-organ failure
OHCA: Out-of-hospital cardiac arrest
ROSC: Return of spontaneous circulation
RRT: Renal replacement therapy
SAPS: Simplified Acute Physiology Score
SOFA: Sequential Organ Failure Assessment
TH: Therapeutic hypothermia
